# Enhanced cardiovascular risk prediction in the Western Pacific: A machine learning approach tailored to the Malaysian population

**DOI:** 10.1371/journal.pone.0323949

**Published:** 2025-06-17

**Authors:** Sazzli Kasim, Putri Nur Fatin Amir Rudin, Sorayya Malek, Nurulain Ibrahim, Xue Ning Kiew, Nafiza Mat Nasir, Khairul Shafiq Ibrahim, Raja Ezman Raja Shariff

**Affiliations:** 1 Cardiology Department, Faculty of Medicine, Universiti Teknologi MARA (UiTM), Shah Alam, Malaysia; 2 Cardiac Vascular and Lung Research Institute, Universiti Teknologi MARA (UiTM), Shah Alam, Malaysia; 3 National Heart Association of Malaysia, Heart House, Kuala Lumpur, Malaysia; 4 Faculty of Medicine, Universiti Teknologi MARA (UiTM), Sungai Buloh Campus, Sungai Buloh, Malaysia; 5 Institute of Biological Sciences, Faculty of Science, University Malaya, Kuala Lumpur, Malaysia; 6 Division of Cardiology, University Malaya Medical Centre (UMMC), Kuala Lumpur, Malaysia; Tehran University of Medical Sciences, IRAN, ISLAMIC REPUBLIC OF

## Abstract

**Background:**

Cardiovascular disease (CVD) is a significant public health challenge in the Western Pacific region, including Malaysia.

**Objective:**

This study aimed to develop and validate machine learning (ML) models to predict 10-year CVD risk in a Malaysian cohort, which could serve as a model for other Asian populations with similar genetic and environmental backgrounds.

**Methods:**

Utilizing data from the REDISCOVER Registry (5,688 participants from 2007 to 2017), 30 clinically relevant features were selected, and several ML algorithms were trained: Support Vector Machine (SVM), Logistic Regression (LR), Random Forest (RF), Extreme Gradient Boosting (XGBoost), Neural Network (NN) and Naive Bayes (NB). Ensemble model were also created using three commonly used meta learners, including RF, Generalized Linear Model (GLM), and Gradient Boosting Model (GBM). The dataset was split into a 70:30 train-test ratio, with 5-fold cross-validation to ensure robust performance. Model evaluation was primarily based on the Area Under the Curve (AUC), with additional metrics such as sensitivity, specificity, and the Net Reclassification Index (NRI) to compare the ML models against traditional risk scores like the Framingham Risk Score (FRS) and Revised Pooled Cohort Equations (RPCE).

**Results:**

The LR model achieved the highest AUC of 0.77, outperforming the FRS (AUC = 0.72) and RPCE (AUC = 0.74). The ensemble model provided robust performance, though it did not significantly exceed the best individual model. SHAP (SHapley Additive exPlanations) analysis identified key predictors such as systolic blood pressure, weight and waist circumference. The study showed a significant NRI improvement of 13.15% compared to the FRS and 7.00% compared to the RPCE, highlighting the potential of ML approaches to enhance CVD risk prediction in Malaysia. The best-performing model was deployed on a web platform for real-time use, ensuring ongoing validation and clinical applicability.

**Conclusions:**

These findings underscore the effectiveness of ML models in improving CVD risk stratification and decision-making in Malaysia and beyond.

## Introduction

Cardiovascular disease (CVD), including coronary heart disease, cerebrovascular disease, and peripheral arterial disease, remains a significant global health challenge. In 2021, CVD affected over 500 million people and caused 20.5 million deaths, accounting for nearly one-third of global mortality [1]. Despite a decline in age-standardized mortality rates, regions such as the Western Pacific, including Malaysia, have witnessed a 47.4% increase in CVD-related deaths, highlighting the persistent burden of these conditions [2]. CVD continues to be the leading cause of non-communicable disease (NCD) deaths, responsible for 44% of the 41 million NCD-related deaths globally in 2019 [3,4].

In response to the growing burden of CVD, the “Regional Action Framework for Noncommunicable Disease Prevention and Control in the Western Pacific” emphasizes the importance of targeted prevention efforts, particularly through screening high-risk individuals. Various risk assessment tools, such as the Framingham Risk Score (FRS), Pooled Cohort Equations (PCE), and WHO/ISH risk charts, have been developed to estimate CVD risk [5–8]. Recent advancements in risk prediction have led to the development of updated tools like SCORE2 and the Revised Pooled Cohort Equations (RPCE), which integrate contemporary population data and refined methodologies [9,10]. However, these tools require thorough external validation before being applied to diverse populations. For example, the WHO CVD risk score, while demonstrating good discriminatory power in a Chinese population, was found to overestimate CVD events, highlighting the necessity for local validation and calibration [11].

Despite these advancements, there remains a critical gap in the availability of an updated CVD risk score specifically tailored for the Asian population. This gap forces clinicians in Asian countries to rely on risk models predominantly developed for Caucasian populations, leading to potential inaccuracies and inconsistent risk stratification, particularly in regions like Malaysia [12]. The development and validation of population-specific risk models are therefore imperative to improving CVD risk prediction and ensuring effective prevention strategies across diverse global populations.

Machine learning (ML) offers a robust alternative to traditional risk-scoring systems by encompassing a range of statistical techniques and algorithms that allow computers to learn from data and improve decision-making without explicit programming. ML algorithms such as Logistic Regression (LR), Support Vector Machines (SVM), Random Forests (RF), Extreme Gradient Boosting (XGB), Naïve Bayes (NB) and Neural Network (NN) have shown significant potential in healthcare applications [13–16]. Unlike traditional statistical methods that often rely on predefined assumptions and models, ML algorithms can adapt and learn from data without stringent assumptions, uncovering hidden patterns and relationships. The implementation of feature selection methods in ML further enhances overall model accuracy [17].

Studies have demonstrated that ML models outperform conventional CVD risk scores like the FRS across various populations. In the United States, a study showed that models like the multi-layer perceptron (MLP) provided better predictive accuracy than traditional methods [18]. Similarly, in the UK, the researcher found that advanced ML models, including Gradient Boosting and AutoPrognosis, outperformed traditional risk scores in predicting CVD events [19]. In Australia, the researcher reported that ML models, such as LR, were more accurate to than the FRS in predicting long-term CVD mortality [20]. Similar trends were observed in Japan [21], China [22] and Korea [23], where ML models consistently outperformed conventional risk scores. These findings suggest that ML-based approaches offer more accurate and personalized risk assessments, potentially leading to improved clinical outcomes.

While ML has demonstrated its ability to outperform conventional CVD risk scores, its full potential in CVD risk assessment remains underexplored, particularly with advanced techniques like stacked ensemble learning (EL) and SHAP (SHapley Additive exPlanations) analysis. Stacked EL is a sophisticated ML approach that combines predictions from multiple models to create a more robust and accurate final model. This technique has proven highly effective in CVD mortality prediction, often surpassing the performance of individual ML algorithms and significantly enhancing patient outcomes [24]. Similarly, SHAP analysis, a powerful interpretability tool, has been employed to provide detailed insights into the contribution of each feature within complex ML models, thereby increasing transparency and trust in ML-driven healthcare applications.

Despite the success of these advanced ML techniques in CVD mortality prediction, their application in CVD risk assessment is still limited. This presents a critical opportunity to leverage these methods to develop models that are not only highly accurate but also interpretable—both of which are crucial in clinical settings. To address this gap, our study will utilize a dataset from Malaysia, a multiethnic country representative of the broader Asian population. By applying feature selection, stacked EL, and SHAP analysis, we aim to develop a CVD risk assessment model that is both precise and interpretable, tailored specifically to the Asian population, and capable of improving risk stratification and clinical decision-making.

## Methods

### Study design and setting

The objective of this study was to address the limitations of current CVD risk scores, which were primarily derived from Caucasian populations, by constructing a model tailored for the Malaysian population. We developed a CVD risk prediction model using data from the Responding to Increasing Cardiovascular Disease Prevalence (REDISCOVER) Study, which is a longitudinal community-based cohort initiated in 2007. This registry contains detailed demographic, clinical, and lifestyle information from a wide range of Malaysian individuals with no prior history of CVD aged 18 and older, recruited between 2007 and 2017. The primary outcomes assessed were fatal and non-fatal CVD events occurring within 10 years after baseline recruitment.

**Fig 1:**
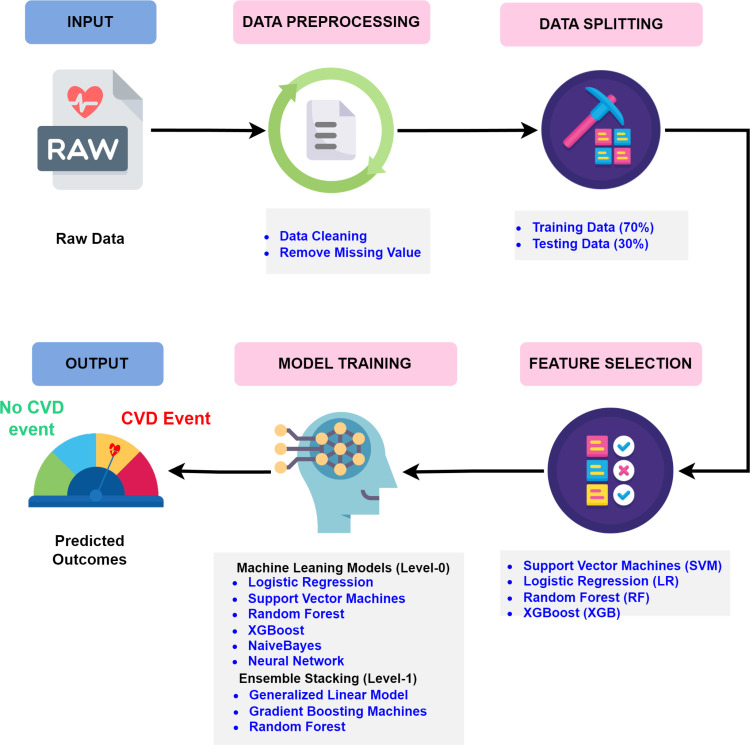
Research workflow and methodology applied in this study.

Participants underwent detailed assessments, and 30 key predictors were selected from an initial set of 202 features. The selected features were applied to construct and evaluate by employing both base and ensemble ML models. The performance of these models was compared against conventional risk scores like the FRS and RPCE to determine the most effective approach for CVD risk prediction in Malaysia. The study’s workflow and methods are shown in Fig 1.

### Data source and study population

The participants in this study were selected from the Responding to Increasing Cardiovascular Disease Prevalence (REDISCOVER) Study, a longitudinal epidemiological study launched by Universiti Teknologi MARA (UiTM) in 2007. The data for this study was collected from the REDISCOVER registry, which spanned the years 2007–2017.

The REDISCOVER study includes a wide range of Malaysian adults aged 18 and up from 40 different communities around the country (18 urban, 22 rural) [25]. Our selection process included a balanced representation of urban and rural areas, with communities chosen from different states and the Federal Territory of Kuala Lumpur in Malaysia. This geographical distribution was designed to reflect the country’s ethnic diversity, with representation from Peninsular Malaysia and Sabah/Sarawak. The study sample was carefully chosen using a systematic four-stage procedure (selection of states, communities, homes, and people within households) to ensure adequate representation of Malaysia’s primary ethnic groups, which included Malays, Chinese, Indians, and indigenous peoples such as the Kadazan-Dusun, Bajau, and Murut of Sabah. States were chosen according to their ethnic composition. The 2000 Malaysian census definition of urban/rural (10,000 people) was employed [26]. A crucial factor for community selection was within-community homogeneity (demographic and socioeconomic), which was essential for the success of our prospective cohort study. Other practical considerations were the necessity of maintaining long-term participant follow-up and building trusting relationships with community leaders.

Participants were recruited with the aid of local community leaders by announcements and written invitations addressed to eligible household members. Participants were required to fast for 8 hours before attending screening sessions at nearby community centres, where their eligibility was assessed. The initial REDISCOVER cohort included 12,617 participants aged 18–89 years, with response rates ranging from 60% to 70% across regions. Each participant provided written informed consent and was monitored every three years, for an average follow-up time of 12.05 years.

From this initial pool of 12,617 cases, a subset of 6,130 cases was selected for this study. The selection criteria required participants to be aged 18 years or older and to have more than 10 years of follow-up data, focusing on individuals recruited between 2007 and 2011. Out of these 6,130 cases, 5688 were identified as complete cases, with no missing values across the variables of interest.

The dataset includes complete details about each participant at the time of recruitment, with additional follow-up data collected every three years. This longitudinal data helped to construct models that can predict CVD outcomes. These models were used to assess CVD risk based on a complete set of the features included in the registry.

For the purposes of this study, ‘current smokers’ were defined as individuals who were either presently smoking cigarettes or had smoked cigarettes within the previous five years. ‘Ex-smokers’ were people who had quit smoking for more than five years, whereas ‘non-smokers’ had never smoked cigarettes.

The REDISCOVER study has been approved by the institutional ethics committee [UiTM Ethics approval number: REC/UITM/2007(10)] and the data usage has been granted for the purpose of the present study by the Project Leader on 14 June 2021. The authors did not have access to information that may be used to identify individual participants during or after the trial. To maintain anonymity, all identifiable data were anonymised before analysis in compliance with ethical requirements.

### Variables and data pre-processing

#### Variables.

This study employed a broad range of variables obtained from the REDISCOVER dataset, encompassing a total of 202 variables at the initial stage. The variables included in this study are classified into the following categories:

- **Demographics:** Age, gender, and area of residence (urban or rural) were included to identify the fundamental traits of the study population.
- **Medical History:** This section related to the participants’ past medical issues, such as hypertension, diabetes, and hyperlipidaemia, which are important for evaluating their risk of CVD.
- **Family Medical History:** Data regarding the prevalence of CVD, hypertension, diabetes, and stroke among close relatives was gathered to consider genetic predispositions.
- **Current Medications:** Information about the usage of medications such as antihypertensives, lipid-lowering agents, and antidiabetics was included to provide insight into the ongoing treatments that may influence cardiovascular outcomes.
- **Anthropometric Measurements:** Variables such as body mass index (BMI), weight, waist circumference, and hip circumference were measured to assess the participants’ physical health and its relation to CVD risk.
- **Clinical Measurements:** This encompasses essential clinical markers such as systolic and diastolic blood pressure, as well as pulse rate.
- **Biochemical Markers:** fasting glucose levels, total cholesterol, triglycerides, HDL, and LDL levels, were used to evaluate metabolic health, which is strongly associated with CVD risk.

The primary outcomes of this study include CVD events that result in death or are non-fatal, and they occur 10 years after the initial assessment. CVD events were carefully documented and evaluated using hospital records and national death certificates at three-year intervals during the follow-up period. The fatal occurrences encompassed myocardial infarction (MI), stroke, congestive heart failure, arterial rupture of an aneurysm, pulmonary embolism, arrhythmic death, death after invasive cardiovascular treatments, and other fatal CVD events. The non-fatal occurrences encompassed periprocedural and non-periprocedural MI, stroke, congestive heart failure, unstable angina, and atrial fibrillation (AF).

#### Data preprocessing.

The data preprocessing steps were essential for ensuring the dataset’s quality and integrity prior to analysis. The following measures were taken:

- **Data Splitting:** The dataset was divided into training and validation sets to create and assess predictive models. Stratified random sampling [27] was utilised to ensure that the outcome variable (CVD events), had similar distributions in both the training (70%) and validation sets (30%). This stage was critical to ensuring that the produced models were robust and generalizable to new data.
- **Data Imputation:** The definition of an incomplete dataset is up to 30% of variables missing. The probability of missing data in our dataset is independent of both observed values and unseen data components. Our dataset is classified as missing completely at random, indicating that the distribution of missing values is random and independent of any variable that may or may not be included in the analysis. We included an additional 442 instances that had missing data. This resulted in a comprehensive dataset that consists of 6,130 patients. This missing case was imputed through multivariate imputation by chained equations, utilizing predictive mean matching from the MICE package in R [28]. This imputation approach estimates missing values by utilizing actual data from similar cases, thus maintaining the integrity of the dataset.
- **Data Balancing:** The dataset demonstrated a class imbalance, with 314 (3.78%) people experiencing cardiovascular disease (CVD) events and 7,997 (96.22%) not experiencing CVD events. For addressing this imbalance, the Random Over-Sampling Examples (ROSE) method was used on the training data [29]. To establish a balanced dataset, this strategy entailed both up-sampling the minority class (CVD events) and down-sampling the majority class (No CVD events). This method was crucial to ensuring that the model did not become biassed in the direction of the majority class and could appropriately predict minority outcomes.
- **Data Normalization:** Continuous variables, such as age, systolic and diastolic blood pressure, and biochemical indicators (e.g., glucose and cholesterol levels), were normalised to reduce the impact of different scales and improve the performance of machine learning models [17]. Standardisation (z-score normalisation) was used to transform these variables into a zero-mean and one-standard deviation distribution.

### Additional statistics

This study includes the mean and standard deviation (SD) for continuous variables, as well as the frequencies and percentages for categorical variables. Correlation analyses were used to investigate the correlations between variables. In order to identify significant categorical variables, the Chi-Square test was used in univariate analysis. For continuous variables, two-sided independent Student’s t-tests were used with a significance threshold of p < 0.001. Additionally, pairwise corrected resampled t-tests were used to compare the performance of the base and ensemble ML models [30,31]. Statistical significance was defined as a p-value of less than 0.001.

### Feature selection

We utilised the sequential backward elimination (SBE) method to identify significant features for our ML models. The SBE algorithm removes irrelevant features in ascending order using model significance value [32]. We began by training four base ML models: SVM with a linear kernel, LR with L1 regularization, RF, and XGB. To determine the relevance of each feature in each base model, we employed the Area Under the Receiver Operating Characteristic Curve (AUC) on a held-out 30% validation set. Specifically, we trained each model using all available features and then calculated the baseline AUC on the validation set. We then retrained the model and recalculated the AUC on the validation set after gradually eliminating one feature at a time. The feature whose removal resulted in the highest AUC on the validation set was considered the least essential and was permanently removed. This procedure was repeated until the best feature subset was selected.

The prediction models were trained and evaluated for each iteration using the 30% validation dataset that was not used for model development. Model performance was assessed using AUC to evaluate discrimination. The models’ predictive performance was calculated, and the models with the highest performance and fewest variables were chosen. Then, the base and ensemble ML models were constructed using the selected features.

### Model development

#### Base ML algorithms.

Several ML techniques were used to develop prediction models for CVD using the R programming language. These algorithms include SVM [33], RF [30], XGB [32], NB [34], LR [35] and NN [36].

The SVM is a robust ML method designed to identify an optimal boundary that maximises the separation between classes. In this study, the SVM model used both linear and Radial Basis Function (RBF) kernel, which is highly advantageous in non-linear classification problems since it transforms the data into a higher-dimensional space that makes it easier to classify. RF algorithm is an EL technique that builds numerous decision trees during training and yields the mode of the classes (classification) or the mean prediction (regression) of the individual trees. RF is known for its reliability and capacity to handle big, high-dimensional datasets, making it resistant to overfitting by averaging numerous decision trees. XGBoost is a part of the gradient boosting family, that generates models in a sequential manner to rectify previous errors. It is highly efficient and scalable, frequently exceeding other ML algorithms in terms of accuracy due to its ability to optimise both the loss function and model complexity through regularisation, making it perfect for large datasets with numerous variables. The NB algorithm is a probabilistic classifier based on Bayes’ Theorem, which implies conditional independence of features given a class label. Despite its simplicity and reliance on feature independence, NB frequently outperforms other classification methods, especially when the independence requirement is met. LR is a well-known statistical method for binary classification that estimates the likelihood of a binary outcome using predictor variables. In this study, we used L1 regularization (Lasso) with LR. L1 regularization improves sparsity in the model by reducing the coefficients of less significant features to zero, hence performing feature selection. Identifying the most relevant risk factors can help improve model interpretability and potentially increase prediction accuracy, which is especially helpful in high-dimensional datasets like ours. NN provide a diverse approach to ML, capable of learning complex nonlinear correlations within data. Their abilities to automatically learn feature characteristics makes them particularly effective when working with complex datasets where feature engineering may be challenging.

The selection of these algorithms was based on prior studies pertaining with cardiovascular disease [23,24,37–40]. A combination of random search and manual adjustment was used to fine-tune the hyperparameters of the base ML and stacked EL models, ensuring that the models are optimally configured for the task.

#### Ensemble ML algorithms.

Stacking, an advanced ensemble ML technique, involves developing a meta-learner to aggregate predictions obtained from various base learners. This strategy takes advantage of the unique features of multiple algorithms, resulting in a model that frequently outperforms any single algorithm in classification and regression tasks. Six notable machine learning algorithms—SVM, RF, XGB, NB, NN and LR—were used as base learners in this study. To develop the final prediction, GLM [41], GBM [42] and RF [30] were utilised as the meta-learner, combining and refining the predictions of the base learners.

5-fold cross-validation was used to avoid overfitting for model development on the training set [43]. While 10-fold cross-validation is typical, we utilized 5-fold cross-validation due to the high computational cost of training multiple complex models on our dataset. This choice provided a practical balance between computing resources and reliable performance evaluation [44,45].

#### Model evaluation.

Model performance was thoroughly evaluated using standardised metrics on an untouched validation dataset [46]. The main metric, AUC, was chosen for its flexibility to assess model performance across a wide range of class distributions, including imbalanced datasets [47,48]. While AUC-PR provides deep insights into performance in minority classes, AUC is still the preferred statistic for overall diagnostic accuracy. In addition to AUC, key metrics including as accuracy, sensitivity, specificity, precision, recall, F1-score, PPV, and NPV were used to ensure a thorough evaluation of the models’ performance. A paired resampled t-test was used as a basis for a more robust comparison of the ML models [49]. Furthermore, the net reclassification index (NRI) was calculated to measure the improvement in correctly classifying both positive and negative cases when the best-performing model was compared to the traditional FRS and RPCE risk scores.

### Results interpretation

Implementing ML models in clinical medicine can be quite challenging because of their complex and black-box nature. Understanding the underlying mechanisms driving predictions can be challenging, as ML models often operate in an agnostic manner [50]. To address this challenge and enhance the interpretability of our models, we chose SHAP (SHapley Additive exPlanations) for our analysis. SHAP values offer valuable insights into how individual input features impact the model’s output, allowing us to determine the significance of each feature and create a ranking system based on their influence [51].

According to Ukwaththa et al. (2024), SHAP offers several key advantages that are particularly relevant in our context [52]. They emphasize the importance of understanding feature contributions and interactions for complex models, a need that is equally critical in clinical prediction. SHAP’s ability to provide both global and local explanations, allows us to understand both the overall behaviour of our model and the specific factors influencing individual patient predictions. This is crucial for building trust and acceptance among clinicians, as it allows them to understand why the model is making specific predictions. Additionally, SHAP’s model-agnostic nature, makes it suitable for our study, which involves comparing and interpreting different model types. By manipulating inputs and closely analysing the resulting predictions, we can gain insights into the behaviour of the model.

### Comparative analysis

Participants in the validation set had been evaluated for their 10-year CVD risk using the FRS and RPCE, which have been previously identified as the most clinically pertinent for the Malaysian population [12]. The specific variables and target outcomes that these risk scores required were employed to implement them. In order to facilitate a direct comparison with the ML models, the accuracy, specificity, sensitivity, AUC, and calibration metrics of these conventional models were calculated.

The DeLong Test was employed to undertake pairwise AUC comparisons between the ML models and the traditional CVD risk scores, with significance determined at a 0.05 level. The optimal high-risk cut-off point of the best-performing ML model was determined to be the threshold at which the sum of sensitivity and specificity was the highest. Subsequently, the NRI was calculated to assess the extent to which the most effective ML model accurately classified both positive and negative cases in comparison to the FRS model. The ML model has the same discriminative power as the FRS model, as indicated by an NRI of zero. In contrast, a negative NRI suggests that the new model is less effective in distinguishing between low-risk and high-risk cases, whereas a positive NRI indicates an improvement in discrimination. A Z-statistic with a significance level of 0.05 was employed to test the null hypothesis (H0: NRI = 0) [53].

### Algorithm deployment

The best-performing model, LR with selected features, along with the FRS algorithm, was deployed on a web platform for real-time use. The algorithm was developed using PHP for server-side scripting, HTML and CSS for front-end design, and SQL for database management. The web platform allows continuous validation and data storage, ensuring the ongoing reliability and accuracy of the model. Users can access the platform to input patient data, receive CVD risk predictions, and compare results with the FRS algorithm, facilitating continuous comparative assessment.

## Results

### Patient characteristics

Table 1 presents the baseline characteristics of the 5,688 participants included in this study. This table provides a univariate analysis of all variables, showing their distribution across the two groups (CVD event vs. no CVD event) and statistical significance.

**Table 1:** Comprehensive baseline characteristics and outcome of the complete dataset.

Variables (n = 30)	Description	All Cases (n = 5688)	CVD Event (n = 202)	No CVD Event (n = 5486)	p-value
**Gender**	1: Male	2400 (42.19%)	121 (59.90%)	2279 (41.54%)	**<0.001**
	2: Female	3288 (57.81%)	81 (40.10%)	3207 (58.46%)	
**Age**		51.82 ± 10.89	58.28 ± 9.89	51.59 ± 10.85	**<0.001**
**Area**	1: Urban	3147 (55.33%)	88 (43.56%)	3059 (55.76%)	**<0.001**
	2: Rural	2541 (44.67%)	114 (56.44%)	2427 (44.24%)	
Menopause	1: Yes	1577 (27.73%)	65 (32.18%)	1512 (27.56%)	0.174
	2: No	4111 (72.27%)	137 (67.82%)	3974 (72.44%)	
**Education**	1: None	821 (14.43%)	42 (20.79%)	779 (14.20%)	**<0.001**
	2: Primary	1361 (23.93%)	76 (37.62%)	1285 (23.42%)	
	3: Secondary	2150 (37.80%)	62 (30.69%)	2088 (38.06%)	
	4: Tertiary	1356 (23.84%)	22 (10.89%)	1334 (24.32%)	
**Smoking**	1: Never	4395 (77.27%)	126 (62.38%)	4269 (77.82%)	**<0.001**
	2: Previous	631 (11.09%)	33 (16.34%)	598 (10.90%)	
	3: Current	662 (11.64%)	43 (21.29%)	619 (11.28%)	
Weight		64.33 ± 13.85	65.79 ± 14.15	64.28 ± 13.84	0.096
Height		1.57 ± 0.09	1.57 ± 0.09	1.57 ± 0.09	0.928
BMI		25.97 ± 4.83	26.66 ± 4.95	25.95 ± 4.82	0.022
**Systolic Blood Pressure**		135.08 ± 22.17	149.00 ± 27.22	134.56 ± 21.80	**<0.001**
**Diastolic Blood Pressure**		80.26 ± 11.80	84.55 ± 15.24	80.10 ± 11.63	**<0.001**
Pulse		75.10 ± 13.09	76.35 ± 13.56	75.05 ± 13.07	0.149
Hip		99.10 ± 10.03	99.64 ± 9.96	99.08 ± 10.04	0.302
**Waist**		85.76 ± 11.83	90.02 ± 11.85	85.60 ± 11.80	**<0.001**
**Diabetes**	1: Yes	623 (10.95%)	49 (24.26%)	574 (10.46%)	**<0.001**
	2: No	5065 (89.05%)	153 (75.74%)	4912 (89.54%)	
**Diabetes** **Medication**	1: Yes	470 (8.26%)	34 (16.83%)	436 (7.95%)	**<0.001**
	2: No	5218 (91.74%)	168 (83.17%)	5050 (92.05%)	
High Cholestrol	1: Yes	694 (12.20%)	25 (12.38%)	669 (12.19%)	1.000
	2: No	4994 (87.80%)	177 (87.62%)	4817 (87.81%)	
Cholestrol Medication	1: Yes	454 (7.98%)	21 (10.40%)	433 (7.89%)	0.247
	2: No	5234 (92.02%)	181 (89.60%)	5053 (92.11%)	
**Hypertension**	1: Yes	1369 (24.07%)	84 (41.58%)	1285 (23.42%)	**<0.001**
	2: No	4319 (75.93%)	118 (58.42%)	4201 (76.58%)	
**Hypertension Medication**	1: Yes	1009 (17.74%)	60 (29.70%)	949 (17.30%)	**<0.001**
	2: No	4679 (82.26%)	142 (70.30%)	4537 (82.70%)	
**Glucose (mmol/L)**		5.54 ± 2.06	6.59 ± 4.00	5.50 ± 1.95	**<0.001**
Total Cholesterol (mmol/L)		5.60 ± 1.19	5.74 ± 1.33	5.59 ± 1.18	0.263
**Triglyceride (mmol/L)**		1.69 ± 1.01	1.97 ± 1.08	1.68 ± 1.01	**<0.001**
**HDL (mmol/L)**		1.25 ± 0.35	1.14 ± 0.33	1.25 ± 0.35	**<0.001**
LDL (mmol/L)		3.58 ± 1.05	3.71 ± 1.17	3.58 ± 1.04	0.279
**Family history of Hypertension**	1: Yes	2289 (40.24%)	51 (25.25%)	2238 (40.79%)	**<0.001**
	2: No	3399 (59.76%)	151 (74.75%)	3248 (59.21%)	
Family history of Diabetes	1: Yes	1513 (26.60%)	42 (20.79%)	1471 (26.81%)	0.069
	2: No	4175 (73.40%)	160 (79.21%)	4015 (73.19%)	
Family history of Heart Disease	1: Yes	1044 (18.35%)	38 (18.81%)	1006 (18.34%)	0.937
	2: No	4644 (81.65%)	164 (81.19%)	4480 (81.66%)	
Family history of Stroke	1: Yes	655 (11.52%)	17 (8.42%)	638 (11.63%)	0.196
	2: No	5033 (88.48%)	185 (91.58%)	4848 (88.37%)	
Family history of Cancer	1: Yes	487 (8.56%)	8 (3.96%)	479 (8.73%)	0.024
	2: No	5201 (91.44%)	194 (96.04%)	5007 (91.27%)	

• The asterisk (*) with p-value < 0.001 indicated that the variable difference between the positive and negative group is statistically significant.

• *Significant values are given in bold and red.*

Among these, 202 (3.55%) had a CVD incident within 10 years of follow-up. The mean age of participants who had a CVD incident was 58.28 (SD 9.9) years, while those who did not have a CVD event were slightly younger, with a mean age of 51.59 (SD 10.85). 64.01% of the patients who had CVD events were male, compared to 42.02% in the non-CVD group. The majority of those who experienced a CVD event lived in rural areas (56.44%) and were current smokers (21.29%), compared to 44.24% and 11.28% in the non-CVD group, respectively. Education levels differed significantly, with 37.62% of CVD event participants having a primary education compared to 23.42% in the non-CVD group. Clinical measures revealed a higher mean BMI (26.66 vs. 25.95), systolic blood pressure (149.00 vs. 134.56 mmHg), and diastolic blood pressure (84.55 vs. 80.10 mmHg) in the CVD group.

In terms of metabolic health, individuals who got CVD had higher glucose levels (6.59 vs. 5.50 mmol/L), higher triglycerides (1.97 vs. 1.68 mmol/L), and lower HDL cholesterol (1.14 vs. 1.25 mmol/L). The CVD group also had a greater incidence of diabetes (24.26% vs. 10.46%) and hypertension (41.58% vs. 23.42%).

Menopause frequency and medication use are presented in S1 Table for subgroups based on diabetes, high cholesterol, and hypertension, further broken down by the occurrence of CVD events. Women with CVD were more likely to be postmenopausal (80.25% vs. 47.15% overall). Across all three conditions, medication use was relatively high: 75.4% of patients with diabetes, 65.4% of patients with high cholesterol, and 73.7% of patients with hypertension were on medication. In the CVD group, medication use was slightly lower for diabetes, but more prevalent for hypertension and high cholesterol.

In conclusion, those who had CVD events were typically older men with higher BMI, blood pressure, glucose, and triglyceride levels. They also had a greater rate of smoking, diabetes, hypertension, and lower HDL cholesterol levels, emphasising the multifaceted nature of cardiovascular disease risk. There were significant differences in gender, age, geographical area, education level, smoking, systolic blood pressure, diastolic blood pressure, waist circumference, diabetes, diabetes medication, hypertension, hypertension medication, glucose level, triglyceride, family history of hypertension, and HDL level for the two groups (p < 0.001).

### Feature selection

SBE feature selection methods were combined with ML algorithms SVM, RF, LR and XGB to identify the most significant predictors among the 30 variables considered, aiming to enhance the performance of CVD predictive models (refer to methods). Table 2 presents a comparison between the rankings of selected features based on variable importance and those used in the FRS and RPCE.

**Table 2:** Comparison between ranked features selected by ML feature selection and the FRS risk score.

FRS Selected Features (8 features)	RPCE features (8 features)	SVM Selected Features (11 features)	RF Selected Features (9 features)	LR Selected Features (12 features)	XGB Selected Features (15 features)
Gender	Gender	**Gender**	**Gender**	**Gender**	**Gender**
Age	Age	Smoking status	Age	Smoking status	Menopause
Smoking status	Smoking status	Weight	Height	Weight	Education
Systolic Blood Pressure	Systolic Blood Pressure	Height	BMI	BMI	Height
Diabetes	Diabetes	Systolic Blood Pressure	Diastolic Blood Pressure	Systolic Blood Pressure	Diastolic Blood Pressure
Total Cholesterol	Total Cholesterol	Waist circumference	**Glucose**	Waist circumference	Pulse
HDL	HDL	Cholesterol medication	**HDL baseline**	Diabetes	Diabetes
	Hypertensive Medication	**Glucose**	Family history of Hypertension	**Glucose**	Diabetes medication
		Total cholesterol	Family history of Cancer	**HDL baseline**	Cholesterol medication
		**HDL baseline**		LDL baseline	Hypertension medication
		Family history of Hypertension		Family history of Hypertension	**Glucose**
				Family history of Cancer	Triglyceride baseline
					**HDL baseline**
					Family history of heart disease
					Family history of Cancer

Notably, gender, HDL, and glucose level emerged as common predictors across all ML feature selection models. Among these, gender and HDL are also integral components of the FRS and RPCE, underscoring their consistent relevance in CVD risk prediction.

### Algorithm performance

On the 30% validation dataset, the ML models built using ML-selected features showed higher predictive performance compared to the FRS and RPCE risk score (Table 3). The best-performing model was the base LR model using LR-selected variables, which significantly outperformed both the FRS and RPCE risk scores (p < 0.001). A detailed performance comparison of the ML models score on a 30% validation set is provided in Table 4. For reference, the performance of the FRS and RPCE risk scores, as well as the ML models with feature selection based on a 70% training dataset, is available in S2 Table.

**Table 3:** The AUC of FRS and RPCE risk scores and ML models with feature selection based on a 30% validation dataset.

Models	The area under the ROC Curve (95% CI)			
	SVM Selected Features	RF Selected Features	LR Selected Features	XGB Selected Features
**Base LR**	0.766 (0.705–0.827)	0.754 (0.694–0.814)	**0.769** **(0.708–0.829)**	0.714 (0.653–0.774)
Base SVM (Linear Kernel)	0.766 (0.706–0.826)	0.752 (0.693–0.812)	0.763 (0.704–0.823)	0.731 (0.671–0.791)
Base SVM (Radial Kernel)	0.559 (0.488–0.63)	0.571 (0.498–0.645)	0.608 (0.542–0.675)	0.516 (0.441–0.591)
Base RF	0.671 (0.604–0.738)	0.756 (0.698–0.813)	0.653 (0.585–0.721)	0.657 (0.597–0.717)
Base XGBoost	0.66 (0.598–0.722)	0.758 (0.694–0.823)	0.682 (0.614–0.75)	0.708 (0.650–0.767)
Base NB	0.721 (0.656–0.785)	0.765 (0.703–0.828)	0.728 (0.661–0.794)	0.703 (0.639–0.766)
Base NN	0.567 (0.490–0.645)	0.617 (0.546–0.689)	0.577 (0.505–0.649)	0.585 (0.511–0.659)
Ensemble ML (GLM meta-learner)	0.605 (0.533–0.677)	0.631 (0.565–0.696)	0.555 (0.476–0.634)	0.560 (0.474–0.646)
Ensemble ML (GBM meta-learner)	0.499 (0.483–0.516)	0.529 (0.486–0.572)	0.485 (0.481–0.489)	0.496 (0.473–0.519)
Ensemble ML (RF meta-learner)	0.669 (0.604–0.735)	0.598 (0.537–0.659)	0.638 (0.564–0.712)	0.654 (0.586–0.721)
FRS	0.716 (0.649–0.783)			
RPCE	0.740 (0.679–0.800)			

**Table 4:** Detailed performance metrics of ML models with feature selection.

Models	Accuracy (95% CI)	Sensitivity	Specificity	PPV	NPV	Precision	Recall	F1-score	Mcnemar’s test (p-value)
**SVM Selected Features**									
Base LR	0.731 (0.710–0.752)	0.650	0.734	0.082	0.983	0.082	0.650	0.146	<0.001
Base SVM (Linear Kernel)	0.730 (0.708–0.751)	0.667	0.732	0.083	0.984	0.083	0.667	0.148	<0.001
Base SVM (Radial Kernel)	0.940 (0.927–0.95)	0.017	0.973	0.022	0.964	0.022	0.017	0.019	0.168
Base RF	0.962 (0.952–0.97)	0.000	0.997	0.000	0.965	0.000	0.000	NA	<0.001
Base XGBoost	0.930 (0.916–0.941)	0.083	0.96	0.071	0.966	0.071	0.083	0.077	0.411
Base NB	0.794 (0.774–0.813)	0.500	0.804	0.085	0.978	0.085	0.500	0.146	<0.001
Base NN	0.806 (0.786–0.824)	0.283	0.825	0.056	0.969	0.056	0.283	0.093	<0.001
Ensemble ML (GLM meta-learner)	0.964 (0.954–0.972)	0.000	0.999	0.000	0.965	0.000	0.000	NA	<0.001
Ensemble ML (GBM meta-learner)	0.964 (0.954–0.973)	0.000	0.999	0.000	0.965	0.000	0.000	NA	<0.001
Ensemble ML (RF meta-learner)	0.965 (0.955–0.973)	0.000	1.000	NA	0.965	NA	0.000	NA	<0.001
**RF Selected Features**									
Base LR	0.690 (0.667–0.712)	0.650	0.691	0.071	0.982	0.071	0.650	0.129	<0.001
Base SVM (Linear Kernel)	0.683 (0.661–0.705)	0.700	0.683	0.074	0.984	0.074	0.700	0.135	<0.001
Base SVM (Radial Kernel)	0.932 (0.919–0.943)	0.050	0.964	0.048	0.965	0.048	0.050	0.049	0.926
Base RF	0.961 (0.951–0.97)	0.033	0.995	0.200	0.966	0.200	0.033	0.057	<0.001
Base XGBoost	0.941 (0.928–0.951)	0.217	0.967	0.194	0.971	0.194	0.217	0.205	0.55
Base NB	0.618 (0.594–0.641)	0.750	0.613	0.066	0.985	0.066	0.750	0.121	<0.001
Base NN	0.787 (0.767–0.806)	0.333	0.804	0.058	0.971	0.058	0.333	0.099	<0.001
Ensemble ML (GLM meta-learner)	0.963 (0.953–0.971)	0.000	0.998	0.000	0.965	0.000	0.000	NA	<0.001
Ensemble ML (GBM meta-learner)	0.962 (0.952–0.971)	0.000	0.998	0.000	0.965	0.000	0.000	NA	<0.001
Ensemble ML (RF meta-learner)	0.965 (0.955–0.973)	0.000	1.000	NA	0.965	NA	0.000	NA	<0.001
**LR Selected Features**									
Base LR	0.730 (0.708–0.751)	0.683	0.732	0.085	0.984	0.085	0.683	0.151	<0.001
Base SVM (Linear Kernel)	0.732 (0.71–0.753)	0.650	0.735	0.082	0.983	0.082	0.650	0.146	<0.001
Base SVM (Radial Kernel)	0.931 (0.918–0.943)	0.083	0.962	0.075	0.966	0.075	0.083	0.079	0.579
Base RF	0.951 (0.94–0.961)	0.050	0.984	0.103	0.966	0.103	0.050	0.067	0.001
Base XGBoost	0.900 (0.884–0.914)	0.150	0.927	0.070	0.968	0.070	0.150	0.095	<0.001
Base NB	0.795 (0.775–0.814)	0.517	0.805	0.088	0.979	0.088	0.517	0.151	<0.001
Base NN	0.825 (0.806–0.842)	0.217	0.847	0.049	0.967	0.049	0.217	0.080	<0.001
Ensemble ML (GLM meta-learner)	0.957 (0.946–0.966)	0.050	0.990	0.158	0.966	0.158	0.050	0.076	<0.001
Ensemble ML (GBM meta-learner)	0.964 (0.954–0.973)	0.000	0.999	0.000	0.965	0.000	0.000	NA	<0.001
Ensemble ML (RF meta-learner)	0.964 (0.954–0.973)	0.000	0.999	0.000	0.965	0.000	0.000	NA	<0.001
**XGB Selected Features (15)**									
Base LR	0.679 (0.656–0.701)	0.600	0.681	0.064	0.979	0.064	0.600	0.116	<0.001
Base SVM (Linear Kernel)	0.660 (0.637–0.682)	0.667	0.660	0.067	0.982	0.067	0.667	0.121	<0.001
Base SVM (Radial Kernel)	0.914 (0.900–0.927)	0.067	0.945	0.043	0.965	0.043	0.067	0.052	0.006
Base RF	0.951 (0.939–0.961)	0.000	0.985	0.000	0.964	0.000	0.000	NA	<0.001
Base XGBoost	0.933 (0.920–0.945)	0.100	0.964	0.091	0.967	0.091	0.100	0.095	0.64
Base NB	0.836 (0.817–0.853)	0.350	0.853	0.080	0.973	0.080	0.350	0.130	<0.001
Base NN	0.840 (0.822–0.857)	0.217	0.863	0.054	0.968	0.054	0.217	0.087	<0.001
Ensemble ML (GLM meta-learner)	0.959 (0.948–0.968)	0.000	0.994	0.000	0.965	0.000	0.000	NA	<0.001
Ensemble ML (GBM meta-learner)	0.964 (0.954–0.973)	0.000	0.999	0.000	0.965	0.000	0.000	NA	<0.001
Ensemble ML (RF meta-learner)	0.964 (0.954–0.973)	0	0.999	0	0.965	0	0	NA	<0.001

The prediction performance of the ML models varied based on the feature selection approach used. Among the individual ML models, the base LR model using features selected by LR demonstrated the highest predictive performance with an AUC of 0.769 (95% CI: 0.708–0.829). Several other base models, particularly those employing LR or SVM for feature selection, achieved comparable performance. However, base models such as SVM (radial kernel) and neural network generally exhibited lower predictive ability. The EL models, designed to combine the strengths of multiple base models, did not consistently outperform the best individual models and, in some cases, performed worse, suggesting that the ensemble approach did not necessarily improve predictive accuracy in this specific context.

When compared to traditional risk rankings, machine learning models produced promising results. The FRS and RPCE risk scores had AUC values of 0.716 and 0.740, respectively. The top-performing ML models, including the base LR with LR-selected features, performed similarly or better than the established risk scores. This shows that ML techniques, particularly when combined with proper feature selection methods, have the potential to improve predictive accuracy beyond what is currently provided by clinical scoring systems such as FRS and RPCE. However, the heterogeneity in performance between ML models and feature selection methodologies highlights the significance of thorough model selection and validation..

### Secondary analysis on best performing model

The best performing ML models, base LR (LR selected var), were also trained on an imputed dataset and evaluated utilizing the 30% complete cases validation dataset. This enables a valid comparison between models constructed with imputed and complete cases models (Table 5).

**Table 5:** Detailed performance metrics of best LR model (LR selected var) on the imputed dataset and complete dataset.

Dataset	AUC (95% CI)	Accuracy (95% CI)	Sensitivity	Specificity	PPV	NPV
Complete Dataset	0.769 (0.708–0.829)	0.730 (0.708–0.751)	0.683	0.732	0.085	0.984
Imputed Dataset	0.768 (0.708–0.828)	0.742 (0.72–0.763)	0.700	0.743	0.091	0.985

LR (LR selected var), trained on imputed datasets performed comparably to models trained on the complete dataset using a similar validation dataset of complete cases: LR (LR selected var) (AUC = 0.768, CI: 0.708–0.828 vs AUC = 0.769, CI: 0.708–0.829) (p = 0.0178). There is no statistically significant difference between the LR model (LR selected var) using complete cases with the imputed model.

### Model interpretation

#### SHAP analysis.

The SHAP summary plots demonstrate the distribution of SHAP values for the significant features identified through LR-based feature selection. These features are used to predict CVD outcomes for Malaysian population.

The vertical axis of each plot displays the features in descending order of their influence on the model’s predictions. The x-axis corresponds to the SHAP value, which measures the influence of each feature on the model’s output. The colour gradient indicates the original value of the feature, with red representing higher values and blue representing lower values.

**Fig 2:**
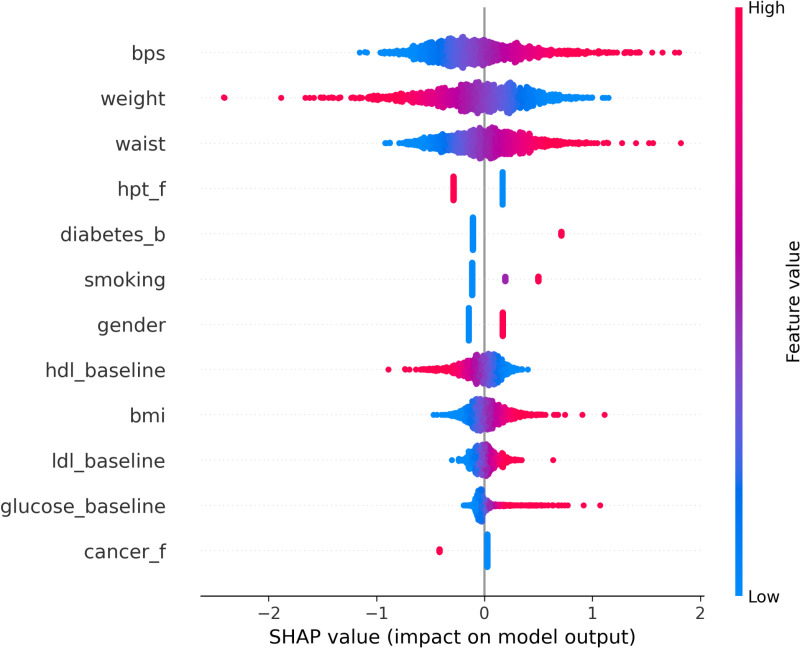
SHAP Analysis.

The SHAP analysis shown in Fig 2 identifies key variables impacting CVD occurrence, demonstrating the direction and extent of each feature’s impact. Systolic blood pressure has the most influence, with higher values (red) closely associated with positive SHAP observations, indicating an increased risk of a CVD event. Weight shows a similar positive association, with increased weight resulting in higher estimated risk. Waist circumference, another important measure of cardiovascular health, follows a similar pattern. A positive family history of hypertension, as well as a diabetes diagnosis, both increase the predicted risk. Baseline HDL cholesterol reveals an inverse connection, with higher HDL levels (red) associated with decreased risk, which is consistent with clinical experience. BMI enhances the information provided by weight and waist circumference. While gender appears to have some influence, it is less significant than the other characteristics. Smoking status has a moderate impact, with current or past smokers having a higher risk. LDL cholesterol and glucose levels have some influence, though not as much as the top-ranked features. A family history of cancer has a relatively minor impact. These variables, among others, highlight the complex nature of CVD risk, which encompasses demographic, clinical, and lifestyle factors.

#### NRI analysis.

NRI for the CVD model, the net reclassification of Malaysian population using the base LR (LR selected var) produced a net reclassification improvement of 13.15% and 7.00 with p < 0.00001 over the original FRS (Table 6) and RPCE (Table 7) risk score respectively.

**Table 6:** NRI Comparison between Machine Learning Methods and FRS.

Individuals with Events (Positive) (n = 60)						
		Number of Individuals		Reclassification		Net Correctly Reclassified (%)
		Machine Learning		Increased Risk	Decreased Risk	-15.00%
		Low Risk	High Risk	5	14	
	FRS Score					
	Low Risk	5	5			
	High Risk	14	36			
**Individuals without Events (Alive) (n= 1645)**						
		Number of Individuals		Reclassification		Net Correctly Reclassified (%)
		Machine Learning		Increased Risk	Decreased Risk	28.15%
		Low Risk	High Risk	67	530	
	FRS Score					
	Low Risk	674	67			
	High Risk	530	374			
Net Reclassification Index (NRI)	-15.00 + 28.15 = 13.15					
Z, p-value	Z= $\frac{13.15}{\sqrt{\frac{5+14\;}{60^{2}}+\frac{67+530}{1645^{2}}\;}}$ = 177.29 177.29, p < 0.00001					
Conclusion	It was statistically significant. ML model has a better predictive ability compared with the FRS risk scores model in predicting the CVD risk of the Malaysian population, and the proportion of correct classification increased by **13.15%.**					

**Table 7:** NRI Comparison between Machine Learning Methods and RPCE.

Individuals with Events (Positive) (n = 60)						
		Number of Individuals		Reclassification		Net Correctly Reclassified (%)
		Machine Learning		Increased Risk	Decreased Risk	8.33%
		Low Risk	High Risk	7	2	
	RPCE Score					
	Low Risk	17	7			
	High Risk	2	34			
**Individuals without Events (Alive) (n= 1645)**						
		Number of Individuals		Reclassification		Net Correctly Reclassified (%)
		Machine Learning		Increased Risk	Decreased Risk	-1.34%
		Low Risk	High Risk	146	124	
	RPCE Score					
	Low Risk	1080	146			
	High Risk	124	295			
Net Reclassification Index (NRI)	8.33 – 1.34 = 7.00					
Z, p-value	Z= $\frac{7.00}{\sqrt{\frac{7+2}{60^{2}}+\frac{146+124}{1645^{2}}\;}}$ = 137.20 137.20, p < 0.00001					
Conclusion	It was statistically significant. ML model has a better predictive ability compared with the RPCE risk scores model in predicting the CVD risk of the Malaysian population, and the proportion of correct classification increased by **7.00%.**					

### Web platform deployment

The developed algorithm was successfully integrated into a web platform, accessible via www.myheartrisk.uitm.edu.my. This platform allows healthcare professionals and researchers to input patient data, receive immediate CVD risk assessments, and compare the ML model’s predictions with those of the FRS algorithm. The platform’s database, managed by SQL, enables continuous data storage and validation, ensuring that the model remains reliable and up-to-date. The use of PHP, HTML, CSS, and SQL in the platform’s development ensures a user-friendly interface and robust functionality.

## Discussion

The findings of this study underscore the effectiveness of ML models in predicting CVD risk, particularly when compared to traditional risk scores such as the FRS and the RPCE. Among the ML models tested, the LR model, especially with LR-selected features, demonstrated the best performance, achieving an AUC of 0.77. This result surpasses the performance of both the FRS and RPCE risk scores, highlighting the potential of ML to enhance predictive accuracy for CVD risk, especially within the diverse Malaysian population.

The superior performance of the LR model can be attributed to several key factors. LR is inherently well-suited for binary classification tasks like predicting CVD events, making it a robust baseline model. The features selected for the LR model, such as gender, systolic blood pressure, HDL cholesterol, glucose levels, and smoking status, were identified using LR and sequential backward elimination methods. These variables are well-established risk factors in the literature [54–56] and play critical roles in cardiovascular health, directly influencing outcomes like blood pressure and lipid metabolism. The inclusion of these features likely contributed to the enhanced predictive power of the LR model, as each plays a significant role in cardiovascular health.

The SHAP analysis conducted in this study provided valuable insights into the key predictors of CVD risk identified by the LR model. The SHAP summary plot illustrates the global contribution of each input feature to the model’s predictions, highlighting the importance of various demographic, clinical, and lifestyle factors in determining CVD risk.

Systolic blood pressure (SBP), weight, and waist size are all significant predictors of CVD risk. Elevated SBP has repeatedly been related to an increased risk of cardiovascular events, with studies indicating that every 10 mm Hg increase in SBP increases the risk of atherosclerotic cardiovascular disease by 53% [57]. This association is continuous and independent, with lower SBP linked with significantly lower cardiovascular mortality [58]. Similarly, weight and waist circumference are key indices of cardiovascular health; studies demonstrate that weight growth and greater waist measurements are associated with an increased risk of coronary heart disease and death. A study discovered that both body mass index (BMI) and waist circumference independently predict higher cardiovascular risk, emphasising the importance of waist circumference in women [59]. Collectively, these variables highlight the necessity of maintaining good blood pressure and body composition in order to reduce CVD risk.

A positive family history of hypertension considerably increases the expected risk of CVD. According to research, family history is an independent predictor of myocardial infarction and stroke, especially in hypertensive individuals [60]. This familial link emphasises the importance of proactive surveillance in at-risk populations. Furthermore, diabetes diagnosis is an important determinant in determining CVD risk due to accompanying metabolic abnormalities. Diabetes has regularly been shown in studies to enhance susceptibility to cardiovascular events; one study found that people with diabetes have a considerably higher risk of CVD events than those without diabetes [61]. This supports the SHAP analysis’s findings, which show that having a family history of hypertension and diabetes increases the predicted risk.

HDL cholesterol has an inverse connection with CVD risk; greater levels are linked to lower risk. A U-shaped connection has been reported, with both low and extremely high HDL levels increasing the risk of cardiovascular events, but moderate levels mitigating against them [62,63]. In contrast, LDL cholesterol is a major contributor to atherosclerosis but has less predictive ability than other indicators such as SBP and metabolic status. Elevated LDL levels have been linked to an increase in CVD mortality, emphasising the importance of efficient lipid management in at-risk individuals [64].

BMI improves our understanding of weight and waist circumference in relation to CVD risk. Although BMI is a common statistic for determining weight status, it might not sufficiently account for the impact of body composition on health outcomes. According to research, BMI corresponds with cardiovascular risk, especially when paired with other measurements such as waist circumference [65]. Gender differences in CVD risk are significant but less pronounced; although men tend to have greater incidences at younger ages, postmenopausal women face increasing risks due to hormonal changes. According to studies, female patients typically have higher cardiovascular risk profiles than males [66].

Smoking status has a major impact on CVD risk, with current or former smokers being more susceptible to cardiovascular events. Numerous studies demonstrate that smoking causes endothelial dysfunction and promotes atherosclerosis, resulting in higher morbidity from heart disease [67]. This emphasises smoking as a significant lifestyle component in CVD prediction.

Glucose levels contribute to CVD risk, however they have lower predictive ability than other variables. Elevated glucose has been associated to diabetes-related issues impacting heart health; however, the direct impact differs depending on individual metabolic profiles. Finally, while a family history of cancer has been identified as a small risk factor for CVD, genetic predispositions can affect a variety of health outcomes [67]. In conclusion, the interaction of these variables demonstrates the multidimensional character of CVD risk assessment. The SHAP study clearly demonstrates how demographic, clinical, and lifestyle factors influence individual cardiovascular health profiles.

The results of this study align with other research conducted in the Asian region, which also emphasizes the effectiveness of ML models over traditional risk scores in CVD risk prediction. For instance, a study from Korea [23] demonstrated that ML models, including neural networks, outperformed the FRS in predicting CVD risk, achieving an AUC of 0.80. Similarly, research conducted in China found that a Random Forest model achieved an AUC of 0.79, surpassing the FRS, which had an AUC of 0.76 [68]. A study from Japan reported that ML models provided better performance in predicting long-term CVD risk compared to conventional methods, with neural networks achieving an AUC of 0.82 [21]. These findings, combined with our study, suggest that ML models are more adaptable to the specific demographic and health characteristics of Asian populations, making them a more suitable choice for CVD risk prediction in these regions.

When comparing the performance of ML models to traditional risk scores in this study, the lower AUC values for the FRS and RPCE (0.74 and 0.76, respectively) suggest that these conventional methods may not fully capture the complexity of CVD risk factors in the Malaysian population. This result is consistent with the findings from other Asian studies, where traditional risk scores have similarly underperformed [23,69]. This underperformance highlights the necessity for developing and validating more population-specific models, such as the ML-based approaches used in this study.

A key strength of this study is the integration of the NRI as an evaluation metric. The NRI value of 13.15% indicates a significant improvement in correctly classifying individuals into appropriate risk categories compared to traditional risk scores, which is crucial for targeted intervention and improving clinical outcomes. The statistical significance of this NRI (p < 0.00001) underscores the practical benefits of using ML models in clinical practice, particularly in multiethnic populations like Malaysia’s, where risk stratification can be complex due to varying genetic, environmental, and lifestyle factors. Notably, the use of NRI in this context is novel, as it has not been widely reported in other studies focused on CVD risk prediction, highlighting the innovation and relevance of this study.

While EL techniques are often expected to outperform individual models, the ensemble models in this study did not exceed the performance of the base LR model. This suggests that while ensemble methods, such as those used in GBM and Neural Networks, can capture complex patterns in data, they may introduce additional complexity that does not necessarily translate into better performance. In contrast, the simpler LR model, combined with effective feature selection and SHAP analysis, provided a balance of accuracy and interpretability that is particularly valuable in clinical settings [28]. The SHAP-enhanced LR model offers a clear understanding of how individual features contribute to the overall risk prediction, making it more actionable for clinicians.

The Malaysian registry, which serves as the basis for this study, is another strength, as it represents a multiethnic population that is reflective of the broader South-East Asian region, including countries like Singapore, Indonesia, India, and China. This diversity enhances the generalizability of the findings, making the ML model applicable across various Asian populations with similar demographic and health characteristics. This broader applicability underscores the potential of the model to be used in different healthcare settings within the region.

This study also highlights the importance of continuous real-world validation, a key strength enabled by the development of an accessible web platform. This platform allows for ongoing validation of the algorithms against real-world data, ensuring that the model remains relevant and accurate as it is applied in clinical practice. Such a resource is critical for maintaining the model’s applicability and improving patient outcomes over time.

Furthermore, the deployment of the ML algorithm on a web platform accessible via www.myheartrisk.uitm.edu.my represents a significant advancement in making these predictive tools accessible to healthcare professionals. This platform also allows for continuous validation against real-world data, which will be crucial in monitoring the model’s performance over time and potentially identifying any drift due to changes in risk factors or population characteristics since the original data collection period. The integration of both the ML model and traditional risk scores like FRS into the platform enables continuous comparative assessment, ensuring the model’s reliability and relevance over time. This approach not only enhances the model’s clinical utility but also supports its adoption in diverse healthcare settings across the Western Pacific region.

A limitation of this study is the use of data acquired between 2007 and 2017. We recognise that lifestyle, healthcare practices, and the prevalence of CVD risk factors may have changed since this time. While this work provides an important baseline and proof-of-concept for the suggested ML models, the applicability of our findings to the current population should be approached with caution. Our data source is actively cleaning and preparing more recent data, which is not yet available for research use. Future research using this updated data will be necessary to test and develop our models, assess their performance in the present environment, and analyse the impact of any changes in CVD risk factors that may have occurred since the initial data.

A key area for future development is the adaptation of our models for shorter prediction horizons. While our study focused on the clinically relevant 10-year risk prediction time frame, we acknowledge that shorter-term projections (e.g., 3-year and 6-year risk) may be more insightful for older adults. Future work will include developing and evaluating models tailored to these shorter timescales.

In summary, the SHAP analysis conducted in this study not only validated the importance of key CVD risk factors but also provided a transparent framework for understanding how these factors influence predictions. This interpretability, combined with the competitive performance of the LR model, positions it as a valuable tool for CVD risk assessment, especially when compared to more complex

## Conclusion

This study demonstrates the potential of ML models, including ensemble learning, to significantly improve CVD risk prediction in the Malaysian population, serving as a model for other countries in the Western Pacific region. The LR model with selected features emerged as the best performer, surpassing traditional risk scores like the FRS and RPCE in predictive accuracy. The incorporation of SHAP analysis provided valuable insights into the most influential predictors, enhancing the interpretability of the ML models.

In addition to developing these predictive models, we successfully deployed the best-performing algorithm on a publicly accessible web platform, allowing healthcare professionals and researchers to apply the model in real-time clinical settings. This platform ensures ongoing validation against real-world data, maintaining the model’s relevance and accuracy over time.

The findings underscore the importance of developing population-specific risk models that account for the unique genetic and environmental characteristics of the Western Pacific region. By demonstrating a significant improvement in the NRI over traditional models, this study highlights the potential of ML approaches to refine risk stratification and support more accurate and personalized clinical decision-making.

This study validates the effectiveness of ML and ensemble learning models in accurately predicting CVD risk within the Malaysian population, offering a model that can be extended to other countries in the Western Pacific region. The deployment of the algorithm on a web platform further enhances its utility, providing a practical tool for real-time clinical decision-making and continuous comparative assessment with traditional risk scores. The platform’s design ensures that the model remains relevant, reliable, and easily accessible, paving the way for improved patient outcomes and public health strategies across the region.

## Supporting information

S1 Table Menopause among Women and Medication Use on Patients with Hypertension, High Cholesterol, or Diabetes. (DOCX)

S2 Table The AUC of FRS and RPCE risk scores and ML models with feature selection based on a 70% training dataset. (DOCX)
