# Supplementary material for: Enhanced cardiovascular risk prediction in the Western Pacific: A machine learning approach tailored to the Malaysian population
Source: PLoS One. 2025 Jun 17;20(6):e0323949. doi: 10.1371/journal.pone.0323949 (PMC12173414; doi:10.1371/journal.pone.0323949)
Supplement: S1 Table — (DOCX) [file pone.0323949.s001.docx]

**S1 Table 1. Menopause among Women and Medication Use on Patients with Hypertension, High Cholesterol, or Diabetes**

| Variable | Subgroup Total | Description | All Cases | CVD Event | No CVD Event |
| --- | --- | --- | --- | --- | --- |
| Menopause (Female only) | 3288 | Yes | 1577 (47.96%) | 65 (80.25%) | 1512 (47.15%) |
|  |  | No | 1711 (52.04%) | 16 (19.75%) | 1695 (52.85%) |
| Hypertension Medication (Hypertensive patient) | 1369 | Yes | 1009 (73.7%) | 60 (71.43%) | 949 (73.85%) |
|  |  | No | 360 (26.3%) | 24 (28.57%) | 336 (26.15%) |
| High Cholesterol Medication (High cholesterol patient) | 694 | Yes | 454 (65.42%) | 21 (84%) | 433 (64.72%) |
|  |  | No | 240 (34.58%) | 4 (16%) | (35.28%) |
| Diabetes Medication (Diabetes patient) | 623 | Yes | 470 (75.44%) | 34 (69.39%) | 436 (75.96%) |
|  |  | No | 153 (24.56%) | 15 (30.61%) | 138 (24.04%) |
