# Supplementary material for: Enhanced cardiovascular risk prediction in the Western Pacific: A machine learning approach tailored to the Malaysian population
Source: PLoS One. 2025 Jun 17;20(6):e0323949. doi: 10.1371/journal.pone.0323949 (PMC12173414; doi:10.1371/journal.pone.0323949)
Supplement: S2 Table — (DOCX) [file pone.0323949.s002.docx]

**S2 Table. The AUC of FRS and RPCE risk scores and ML models with feature selection based on a 70% training dataset**

| **Models** | **The area under the ROC Curve**  **(95% CI)** | | | |
| --- | --- | --- | --- | --- |
|  | **SVM Selected Features** | **RF Selected Features** | **LR Selected Features** | **XGB Selected Features** |
| Base LR | 0.74  (0.725 - 0.755) | 0.738  (0.722 - 0.753) | 0.745  (0.73 - 0.76) | 0.732  (0.716 - 0.747) |
| Base SVM  (Linear Kernel) | 0.742  (0.727 - 0.757) | 0.739  (0.724 - 0.755) | 0.748  (0.733 - 0.763) | 0.735  (0.72 - 0.751) |
| Base SVM  (Radial Kernel) | 0.940  (0.931 - 0.949) | 0.929  (0.919 - 0.939) | 0.934  (0.926 - 0.943) | 0.928  (0.918 - 0.938) |
| Base RF | 0.971  (0.965 - 0.976) | 0.97  (0.965 - 0.975) | 0.967  (0.961 - 0.973) | 0.968  (0.963 - 0.973) |
| Base XGBoost | 0.930  (0.921 - 0.938) | 0.892  (0.882 - 0.902) | 0.859  (0.848 - 0.871) | 0.930  (0.921 - 0.938) |
| Base NB | 0.761  (0.746 - 0.775) | 0.764  (0.75 - 0.779) | 0.756  (0.741 - 0.771) | 0.756  (0.741 - 0.771) |
| Base NN | 0.871  (0.859 - 0.883) | 0.884  (0.872 - 0.895) | 0.898  (0.887 - 0.909) | 0.889  (0.879 - 0.9) |
| Ensemble ML (GLM meta-learner) | 0.974  (0.969 - 0.979) | 0.978  (0.974 - 0.982) | 0.972  (0.967 - 0.977) | 0.973  (0.968 - 0.977) |
| Ensemble ML (GBM meta-learner) | 1.000 (1.000 - 1.000) | 1.000 (1.000 - 1.000) | 1.000 (1.000 - 1.000) | 1.000 (1.000 - 1.000) |
| Ensemble ML (RF meta-learner) | 1.000 (1.000 - 1.000) | 1.000 (1.000 - 1.000) | 1.000 (1.000 - 1.000) | 1.000 (1.000 - 1.000) |
| FRS | 0.716 (0.649 - 0.783) | | | |
| RPCE | 0.740 (0.679 - 0.800) | | | |
